# Rosmarinic acid prevents fibrillization and diminishes vibrational modes associated to β sheet in tau protein linked to Alzheimer’s disease

**DOI:** 10.1080/14756366.2017.1347783

**Published:** 2017-07-13

**Authors:** Alberto Cornejo, Felipe Aguilar Sandoval, Leonardo Caballero, Luis Machuca, Patricio Muñoz, Julio Caballero, George Perry, Alejandro Ardiles, Carlos Areche, Francisco Melo

**Affiliations:** a Escuela de Tecnología Médica, Facultad de Medicina, Universidad Andres Bello, Santiago, Chile;; b Departamento de Física, Facultad de Ciencias Fisicas y Matematicas, Universidad de Chile, Santiago, Chile;; c Centro bioinformático y modelamiento molecular, Facultad de Ingeniería, Universidad de Talca, Talca, Chile;; d College of Sciences, University of Texas at San Antonio, San Antonio, TX, USA;; e Departamento de Química, Facultad de Ciencias, Universidad de Chile, Santiago, Chile

**Keywords:** Alzheimer’s disease, tau, aggregation, β-sheet, inhibition, pharmacophore

## Abstract

Alzheimer’s disease is a common tauopathy where fibril formation and aggregates are the hallmark of the disease. Efforts targeting amyloid-β plaques have succeeded to remove plaques but failed in clinical trials to improve cognition; thus, the current therapeutic strategy is at preventing tau aggregation. Here, we demonstrated that four phenolic diterpenoids and rosmarinic acid inhibit fibrillization. Since, rosmarinic acid was the most active compound, we observe morphological changes in atomic force microscopy images after treatment. Hence, rosmarinic acid leads to a decrease in amide regions I and III, indicating that rosmarinic acid prevents β-sheet assembly. Molecular docking study inside the steric zipper model of the hexapeptide ^306^VQIVYK^311^ involved in fibrillization and β sheet formation, suggests that rosmarinic acid binds to the steric zipper with similar chemical interactions with respect to those observed for orange G, a known pharmacofore for amyloid.

## Introduction

Alzheimer’s disease (AD), the most common form of dementia, is characterized by the deposition of two main protein aggregates in the brain: senile plaques consisting of amyloid-β (Aβ) and neurofibrillary tangles (NFTs) composed mainly of the microtubule-associated protein tau and found as intraneuronal inclusion^1^. Tau protein also plays an important role in other tauopathies, such as progressive nuclear palsy (PSP), corticobasal degeneration (CBD), and Pick's disease. In these tauopathies, tau is located in neurons and glia^2^^,^^3^. Physiologically, tau regulates microtubule stability and axonal transport^4^, but in patients with AD it becomes increasingly phosphorylated. Hyperphosphorylated tau no longer binds to microtubules and accumulates in the soma and dendrites of neurons, forming intracellular deposits called paired helical filaments (PHFs) that are the main fibril component of NFTs^5^^,^^6^. Tau pathology occurs concomitantly with axonal transport deficits and neuronal degeneration^7^.

On the other hand, human tau is a natively unfolded protein whose structure has two fibril-forming motifs, ^275^VQIINK^280^ and ^306^VQIVYK^311^. These motifs are within the microtubule-binding domain (4R) of tau (aa 244–373), and are frequently used to resemble tau aggregation *in vitro*^8^^,^^9^. Crystallization of the fibril-forming motifs shows that they are both necessary and sufficient for tau fibrillization. In particular, the glutamine residue in the ^306^VQIVYK^311^ hexapeptide is essential for tau fibrillization^8^^,^^10^. The hexapeptide ^275^VQIINK^280^ is of equal importance for tau fibril formation; unfortunately no crystal structure is available^11^. The protein secondary structure with the highest tendency to form fibrils are β sheets that can associate adjacent strands running either parallel or antiparallel^8^. The ^306^VQIVYK^311^ hexapeptide forms parallel β sheets that are linked through interdigitating non-polar side chains^12^. The correlation of pathological phosphorylation of tau triggers conformational changes that make it prone to form dimers or higher order oligomers^13^^,^^14^. Interestingly, improvement in the cognition of a transgenic mouse model displaying both NFTs and senile plaques required reduction of soluble tau but not of Aβ plaques^15^. Over-expression of tau in neurons has deleterious effects, suggesting that intracellular tau aggregates are cytotoxic^16^.

The major focus of past drug developments for AD treatment has rested on Aβ, although a significant number of candidate drugs have failed to slow down disease progression. For example, immunization against Aβ was effective in reducing amyloid plaque load, but had little effect on cognitive functions in patients^17^^,^^18^. Moreover, a recent study shows that gamma-secretase inhibition failed in a clinical phase III trial^19^. Thus, it seems timely to consider alternate drug discovery strategies for AD, based on approaches such as misfolded tau reducing agent^20^. The development of small-molecules that inhibit aggregation of tau appears as a valid alternative strategy for developing new treatments for AD and other tauopathies^21^. It has been proposed that naturally occurring phytochemicals have the potential to prevent AD based on their neuroprotective, anti-amyloidogenic, anti-oxidative and anti-inflammatory properties^22^. For instance, natural polyphenols have reported anti-aggregating capacity to prevent amyloid formation,^23–25^ and a standardized turmeric extract reduced both amyloid-β and phosphorylated tau levels in a transgenic mouse model^26^. Likewise, novel compounds have been found with the ability to prevent tau aggregation, such as cinnamon extract, and fulvic acid among others^27^^,^^28^.

*Rosmarinus officinalis* L. belonging to Lamiaceae family has been used in medicine as anti-inflammatory and antimicrobial agent^29^^,^^30^. Since drugs targeting Aβ have recently failed in clinical trials^31^^,^^32^, it seems timely to search for other naturally occurring polyphenols with relevant activities, such as tau inhibitors. Considering that tau pathology involves not only AD but several tauopathies, we investigated the effect of four phenolic diterpenes and one caffeoyl derivative, isolated from *Rosmarinus officinalis* L., over heparin-induced tau aggregation *in vitro* by using thioflavin T assay (ThT), atomic force microscopy (AFM), and Raman spectroscopy. In addition, we provide structure-based models of complex between the fibril-forming motif ^306^VQIVYK^311^ of tau and caffeoyl derivative to explain the mechanism of its tau anti-aggregation capacity.

## Materials and methods

### Chemistry

Silica gel (Kieselgel 60, Merck 0.063–0.200 mm) and Sephadex LH-20 were used in column chromatography (CC). Technical solvents used in extraction were previously distilled and dried according to standard procedures. The extraction and purification of **1,2,3,4,** and **5** were carried out essentially as described^33–36^. The purity of all compounds was estimated by using a HPLC-PDA (≥ 98%) (Agilent LC-1200). Tau 4R (four microtubule binding domain) recombinant protein was expressed in *E coli.* The expressed protein was purified by HPLC using a ProPac IMAC-10 Column. Aggregation and fluorescence assays were performed in a Biotek H1 monochromator-based multi-mode microplate reader. Samples were prepared on highly ordered pirolytic graphite (HOPG) as substrate, and atomic force images were obtained using a Nanoscope III equipment in tapping mode. Raman spectra and maps were obtained through a Confocal Raman Microscopy Alpha 300 (WITec GmbH) .

### General experimental procedures of extraction and isolation

*Rosmarinus officinalis* L. was collected in Talca, VII Región, Chile. Samples from aerial parts were dried at room temperature in darkness. *R. officinalis* L. was identified by Prof. O. Garcia, and a voucher specimen (N° RO-2015/1215) is kept at the Herbarium of Extreme Natural Product Laboratory of University of Chile.

Dried and pulverized leaves of *R. officinalis* L. (1000 g) were extracted at room temperature using methanol (3 times, 3 L each time). The resulting solution was evaporated under vacuum to yield 65 g of extract, which was subjected to Sephadex LH-20 (4 times, 10 × 100 cm) using methanol as mobile phase giving four fractions A–D. The only fractions studied were of medium polarity (C–D). Fraction C (15 g) was subjected to column chromatography on silica gel (12 × 90 cm, 63–200 μm) with mixtures of hexane containing 10% increments of ethyl acetate to yield 9 fractions. A crude mixture of phenolic diterpenoids was detected on TLC (Liebermann reactive) eluting with 50% ethyl acetate in hexane (fraction 5). After repeated CC on silica gel of Fr 5, the phenolic diterpenes carnosol (**1**) (2 g), carnosic acid (**2**) (800 mg), rosmanol (**3**) (8 mg), and the mixtures of rosmanol **3** and epiisorosmanol (**4**) were isolated. Compounds **3** (6 mg) and **4** (9 mg) were further purified by a preparative RP-HPLC (PrepCHROM C-700, BÜCHI) using as mobile phase H_2_O–MeOH (9:1,8:2, 7:3, 6:4; 5:5; 4:6, 3:7, 2:8, 1:9).

Fraction D (5 g) was fractionated by CC on silica gel (5 × 60 cm, 63–200 μm) eluted with methanol in dichloromethane giving 3 fractions (A–C). Fractions of interest were monitored by DPPH (5% in MeOH). Some 200 mg of fraction C were further purified by a preparative RP-HPLC using as mobile phase H_2_O–MeOH (9:1, 7:3, 5:5; 3:7, 1:9) to give rosmarinic acid (**5**) (150 mg).

### Protein expression and purification of tau 4R

The fragment 4R (htau_244–372_) was cloned into pET-28a vector (Novagen) to produce a His-tagged protein which was expressed in *Escherichia coli* strain BL21 (DE3) as described^28^. LB medium containing kanamycin (30 μg/ml) was inoculated with a stationary overnight culture. The culture was grown at 37 °C to OD_600_ of 0.5–0.6, and protein expression was induced by addition of 1 mM isopropyl β-D-1-thiogalactopyranoside (IPTG) for 4 h. The cells were pelleted and then resuspended in 50 ml of buffer A (50 mM sodium phosphate buffer pH 7.4, 500 mM NaCl, and 20 mM imidazole) supplemented with EDTA free anti protease tablet (Roche). The mixture was sonicated for 5 min, centrifuged at 10000 g for 30 min and then filtered (0.22 μm membrane filter, Millipore). The sample was loaded onto ProPac IMAC-10 Column (4 × 250 mm Thermo dionex) with buffer A and buffer B (50 mM sodium phosphate buffer pH 7.4, 500 mM NaCl, 200 mM imidazole).

After loading, one-step wash with 30 mmol Imidazole (7.7% B) was done before elution at 200 mmol imidazole. Protein purity was verified on Coomassie Brilliant Blue-stained SDS-polyacrylamide gel. The concentration of purified 4R was determined using the extinction coefficient at 280 nm (1520 M^−1 ^cm^−1^). The concentrated protein was stored at –80 °C until use.

### Tau fibrillization *in vitro*

The aggregation of 4R was induced with 40 μM of protein plus 10 μM of heparin in 100 mM of sodium acetate pH 7.0 for 24 h incubation time, with continuous shaking. Standard solution of **5** at 10 mM to 1 mM (DMSO) was prepared fresh prior to aggregation experiments. We used a black flat bottom 96 well plate (ThermoFisher scientific).

### Atomic force microscopy images of tau protein

Each sample was prepared by pipetting 30 μL (40 μM of tau plus 10 μM of heparin) in the presence or absence of **5** (10 μM and 1 μM respectively) onto highly orientated pyrolytic graphite (HOPG). The samples were incubated at room temperature for 30 min and then carefully washed. Atomic force microscopy imaging was performed in tapping mode using a Nanoscope III (Veeco, CA) and Olympus AC240TS cantilevers exhibiting spring constants of 2 N/m at resonance frequencies of 55 kHz. To achieve minimal imaging forces between AFM stylus and sample, the drive amplitude was set between 0.5 and 1.0 V, and the amplitude set point was adjusted manually to compensate for the thermal drift in order to maintain good tracking of the sample.

### ThT binding fluorescence assay of tau protein

The ThT fluorescence assay adopted here was modified from the reported by Pickhardt et al.^37^ and Crowe et al.^38^. Briefly, to examine the inhibition of tau aggregation, the total volume of the reaction mixture was 100 μL, which included 40 μM of protein, 10 μM heparin in 100 mM sodium acetate, pH 7.0 plus **5** at different concentrations. Notice that for inhibition all components are included in the solution at the same time. After 24 h incubation time at 37 °C, addition of 100 μL at a 50 μM solution of ThT was made and incubation continued for 1 h at room temperature prior to fluorescence reading. Then, the fluorescence was measured in a BioTek Synergy H1 multimode-multiple reader with an excitation wavelength at 440 nm and emission wavelength of 485 nm in a 96-well plate.

### Raman spectra and maps of tau β sheets content

The Raman spectra of 4R were recorded as described with modifications by using an Confocal Raman Microscope Alpha 300 (WITec GmbH) equipped with a solid state Compass Sapphire laser (Coherent Inc.) operating at 532 nm and a Back Illuminated CCD camera DV401A (Andor Technology Ltd.) cooled at −60 °C^39^. A single mode 3.5/125 μm optical fiber was used (OZ Optics Ltd.) to connect the laser to a Zeiss (100X/0.9 NA) objective. The laser power was set between 10 to 20 mW. In order to generate scan images, we set 20 points per line over a surface of 5 μm (width) and 5 μm (height). Scan speed was set to 100 s/line. Integration time was set to 5 s. Tau protein sample (40 μM plus 10 μM of heparin) over glass (microscope slides) was dried and the spectra collected on several spots over the sample. The sample spectra were standardized from glass (spectra subtracted)^39^.

### Molecular docking of ^306^VQIVYK^311^ fragment of tau and 5

Docking was performed by using the Glide method^40^^,^^41^. A grid box of 15 Å × 10 Å × 10 Å covered the whole cavity in the model. Docking parameters were used as in previous work^39^, and Glide extra-precision (XP) modes were explored during the search. The docking hierarchy begins with the systematic conformational expansion of the ligand followed by placement in the receptor site. Then minimization of the ligands (R and S enantiomers) in the receptor field were carried out using the OPLS-AA force field with a distance-dependent dielectric of 2.0^42^. Afterwards, the lowest energy poses were subjected to a Monte Carlo procedure that samples the nearby torsional minima. The best pose for a given ligand was determined by the Emodel score, while different compounds were ranked using GlideScore^43^. The docking poses were analyzed by examining their relative total energy score. The more energetically favorable conformations were selected as best poses.

### Statistical analysis

Statistical analysis was developed using Graphpad 6. Data were analyzed as Mean ± SE using Paired *t*-test and Anova. Significance was determined as *p* < 0.05.

## Result and discussion

Phenolic diterpenes and caffeoyl derivative inhibit fibrillization of tau monitored by ThT fluorescence assay

Interestingly, some phenolic diterpenoids such as carnosic acid has been reported as a potent activator of a nuclear factor erythroid 2 related transcription factor (Nrf-2), a major regulator of redox homeostasis in mammals, whose activity relies on a cathecol moiety^44^^,^^45^^.^ Also phenolic diterpenoid carnosic acid has shown effects on Aβ peptides production in a cells model^46^^,^^47^. Another phenolic diterpenoid, carnosol, has been reported as an anti-inflammatory and anti carcinogenic agent, and blocked inactivation of muscarinic acetylcholine receptor^48–50^.

**Figure 1.**
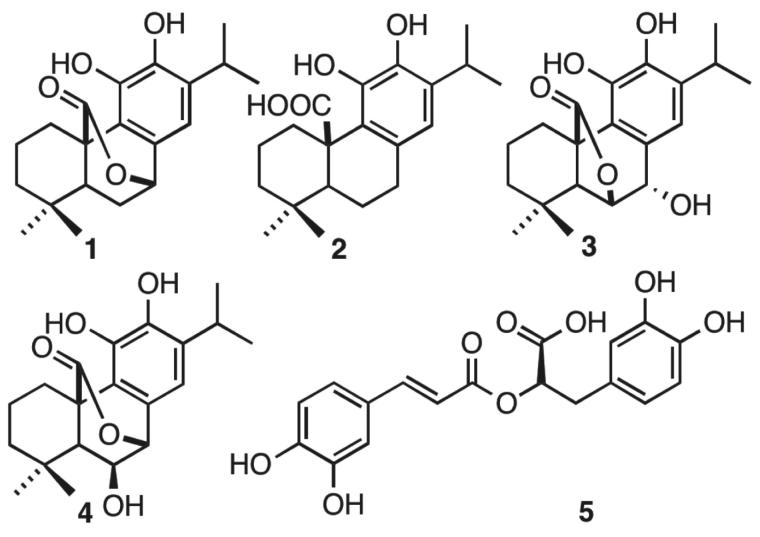
(a) Structure of **1**, **2**, **3**, **4**, and **5** isolated from *Rosmarinus offcinalis* L.

**Figure 2.**
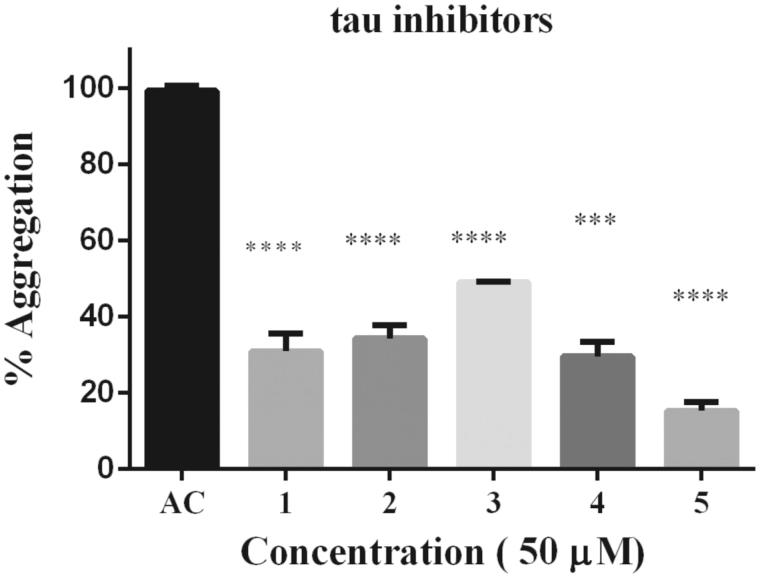
Thioflavine ThT assay over tau fibrillization. (A) Four phenolics diterpenoids, **1**, **2**, **3**, **4**, and a caffeoyl derivative **5** were challenged against tau aggregation at 50 μM. All compounds were active as inhibitors against tau aggregation. Data were presented as mean ± SEM and analyzed using ANOVA. Significance was set as *p* < 0.05.

**Figure 3.**
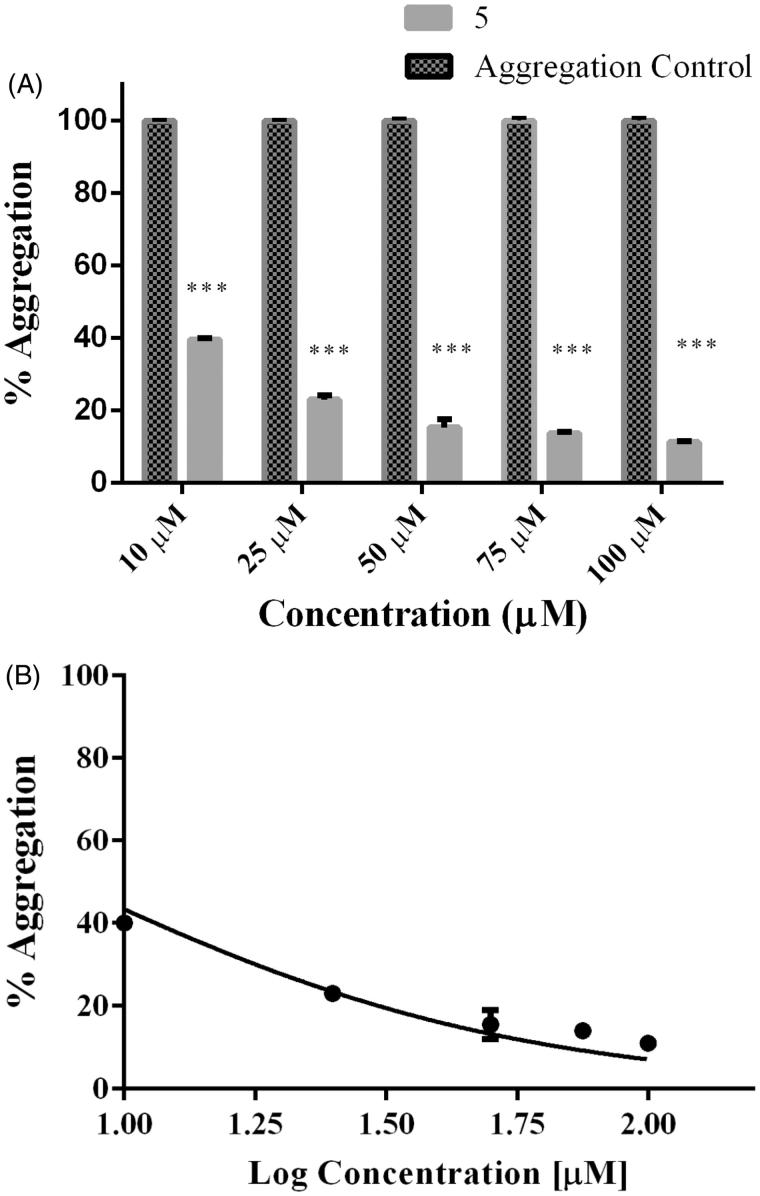
Dose–response effect of **5** over tau 4R aggregation process monitored by ThT. Serial concentration of **5** between 10 μM and 100 μM. B) To obtain IC_50_ the curve fit method was set as non-linear regression. IC_50_ calculated correspond to 7.7 μM. Data were presented as mean ± SEM and analyzed using paired *t*-test showed there was a significant differences between control and treatment conditions t (8) = 15,406, *p* < 0.05.

Moreover, rosmarinic acid isolated from the spice sage (*Salvia officinalis*) protects PC12 cells from neurotoxicity induced by amyloid-β^51^ and has protective effects on transgenic mice carrying a double mutation linked to an amyloid precursor protein^52^. However, until now neither phenolic diterpenoids, nor **5** have been tested against tau aggregation. Thus, we isolated **1, 2, 3, 4,** and **5** from *Rosmarinus officinalis* L., in order to demonstrate whether they may act as tau inhibitors by avoiding fibril formation *in vitro* and subsequent β sheet formation. The chemical structure of phenolic diterpenoids is shown in Figure 1. The ^1^H-NMR spectrum of **1**, **2**, **3**, **4**, and **5** is consistent with the literature^32–35^. Tau protein largely exists as an intrinsically disordered protein^45^, however as long as motifs ^275^VQIINK^280^ and ^306^VQIVYK^311^ interact, tau is able to form paired helical filaments with cross β-sheets^8^. In addition, the benzothiazole dye, ThT, has been used as a fluorescence marker for amyloid fibrils;^53^ giving a reliable signal to determinate fibril content *in vitro*. Therefore, we address the question of whether both phenolic diterpenoids and **5** are able to stop aggregation of tau by carrying out ThT fluorescence assay as an indicator of fluorescence intensity associated to aggregation. Thus, we demonstrate that all compounds are able to inhibit tau aggregation at 50 μM (Figure 2(A)). Considering the inhibition percentage, all naturally occurring compounds inhibit over 50%, and the most active compound was **5** (Table 1). Since **5** was the most effective compound, supplemental assays are performed in order to provide understanding of how **5** interacts with tau. First, we induced tau fibrillization and then we added **5** at different concentrations, after incubation we measured fluorescence intensity. Thus, we found that **5** inhibits tau in concentrations ranging from 10 μM to 100 μM, in dose-response manner (Figure 3(A)). In addition, we calculated IC_50_ at 7.7 μM of **5** based on a nonlinear regression method (Figure 3(B)).

**Table 1.** Table of Inhibition (%) among phenolic diterpenoids (**1**,**2**,**3**,**4**) and caffeoyl derivative (**5**). All compounds inhibit over 50%, including the most active compound **5** (84%).

Natural compounds	Inhibition (%)
**1**	68
**2**	65
**3**	50
**4**	70
**5**	84

It is important to note that nitrocatechol derivatives such as tolcapone and entacapone exhibit anti-aggregation properties against both Aβ and α-synuclein and tau hexpeptide^54–55^.

Considering that **5** was the most active compound and it has two cathecol moieties, we hypothesize that differences observed can be evidence of a main role of cathecol moiety.

### Compound 5 diminishes fibril formation and reduces band assignment modes associated to β-sheets

**Figure 4.**
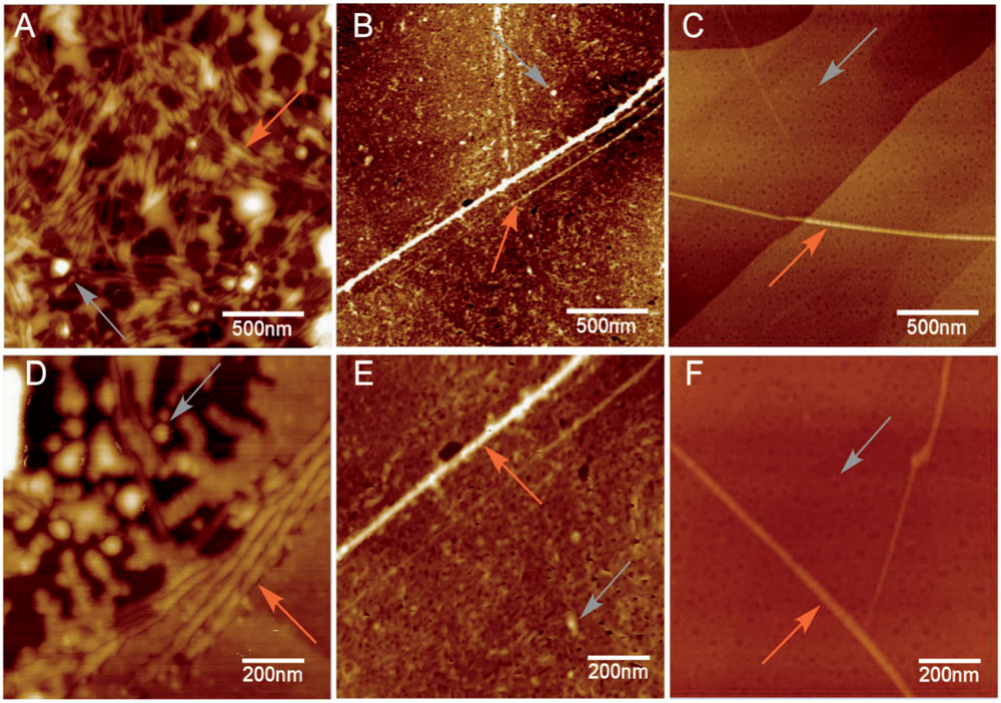
Atomic force microscopy. Height images of 4R aggregation process in absence (positive control) and presence of **5**, at two distinct magnifications. (A) and (D) Positive control (4R aggregation). (B) and (E) 4R aggregation in presence of **5** at 1 μM. (C) and (F) 4R aggregation in presence of **5** at 10 μM. Both gray and orange arrows represent oligomers and fibrils respectively.

**Figure 5.**
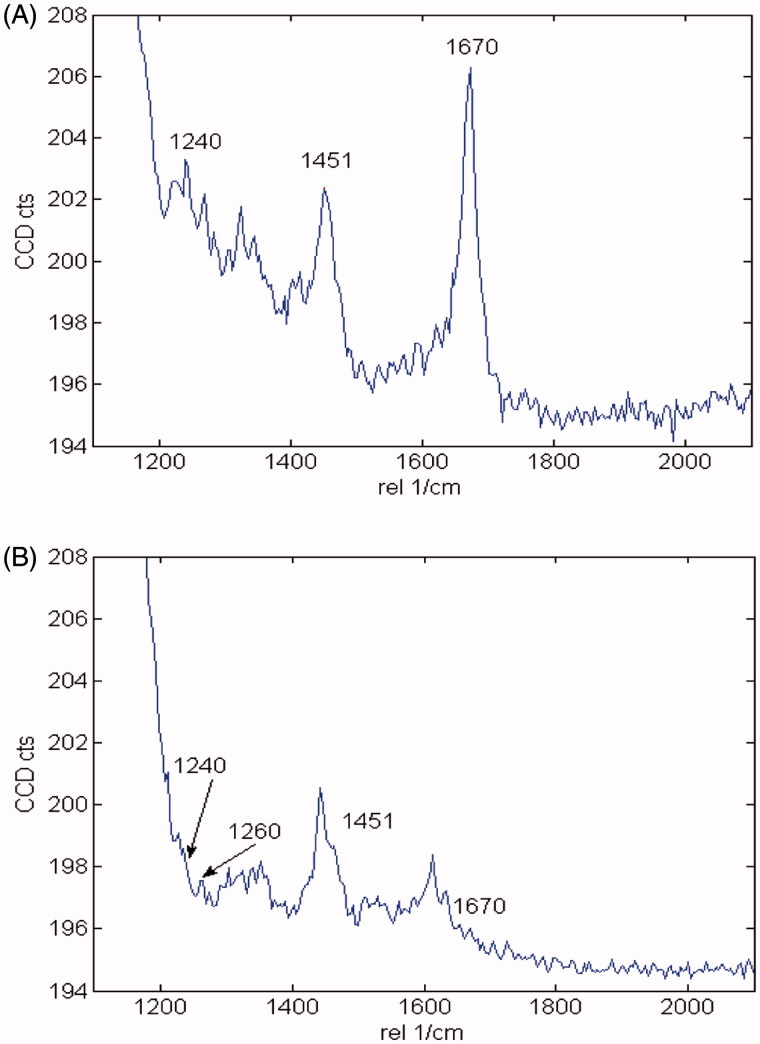
Raman spectra of 4R after aggregation induction in both the absence and presence of **5**. (A) Raman spectra of tau aggregation at 532 nm absence of **5**. Band assignments correspond to Amide I (1670 cm^−1^), Amide III (1240 cm^−1^) and C–H deformation (1451 cm^−1^). (B) Raman spectra of tau aggregation at 532 nm in presence of **5**. Band assignments correspond to Amide I (1670 cm^−1^), Amide III (1240 cm^−1^) and C–H deformation (1451 cm^−1^), Amide III (1260 random coil).

The hallmark of Alzheimer´s disease and tauopathies is the formation of paired helical filaments and subsequent fusion into neurofibrillary tangles (NFTs) whose core is mainly composed of β-sheets structure^56^. Moreover, kinetic studies of fibril formation by using ultraviolet resonance Raman (UVRR) spectroscopy have revealed different stages in fibrillation where at early stages it is possible to find fibrillary aggregates with β sheet and disordered content. As the process advances, the β sheet content also increases^57^. Therefore, there is a correlation between Raman spectroscopy, band assignment, and secondary structure^58^. The sensitive Amide I region is associated with C=O stretch and is able to form a hydrogen bond with an N–H group of either inter- or intra-backbone chain giving a good signature of a β-sheet between wavenumbers 1674–1670 cm^−1^. Moreover, Amide III region is associated to C–N stretching and N–H bending giving a β-sheet signature in the range of wavenumber 1242–1227 cm^−1^. Hence, Raman spectroscopy provides a useful method to monitor β sheet formation^59^. Considering that **5** was the most active naturally occurring compound that we isolated from *R*. *officinalis*, we elucidate whether there are both morphological and signature changes in secondary structure once **5** interacts with tau protein. With this in mind, we induce tau aggregation by adding heparin, which promotes paired helical fibril formation. Atomic force microscopy images (Figure 4) show several twisted fibrils and oligomers at two distinct magnifications. Aggregation control experiments are presented in Figure 4(A) and in Figure 4(D) with higher magnification. Interestingly, once we challenge tau aggregation with **5** at 1 μM, a few straight-thin filaments and several oligomers are seen on the HOPG surface (Figure 4(B,E)). However, at 10 μM of **5** most fibrils and oligomers are diminished indicating that **5** is able to prevent fibril progression (Figure 4(C,F)). Raman’s spectra of tau aggregation (24 h) and Raman vibrational bands are both shown in Figure 5(A), and the vibrational modes are listed in Table 2. Figure 5(A) shows band assignments associated to Amide I and Amide III that are linked to β sheets. In addition, we found band assignment associated to C–H deformation at 1451 cm^−1^ (Figure 5(A)). However, the spectra of tau aggregation in the presence of **5** (10 μmol) (Figure 5(B)), clearly shows a band assignment reduced in both Amide I and III region, respectively, and a signal corresponding to disordered structure at 1260 cm^−1^ ^38^^,^^57–60^ is also detected, indicating that **5** prevents fibrillization and diminished β-sheet content. Considering that tau is a natively unfolded protein,^55^ this suggests that some tau protein at least remains in the disordered state (Figure 5(B)), differently from tau in absence of **5**, where we found a massive peak at 1670 cm^−1^ indicative of β sheet antiparallel structure. Interestingly, inhibitors with a nitrocathecol moiety are able to prevent aggregation and β-sheets assembly as demonstrated by circular dichroism^55^.

**Table 2.** Raman Vibrational bands of tau (4R) associated to secondary structure.

Bad frequency (cm^−1^)	Amide bands	Vibrational mode	Secondary structure
1670	Amide I	C=O stretch	β-Sheet
1240	Amide III	N–H bending and C–N stretching	β-Sheet
1260	Amide III	N–H bending and C–N stretching	Disordered

**Figure 6.**
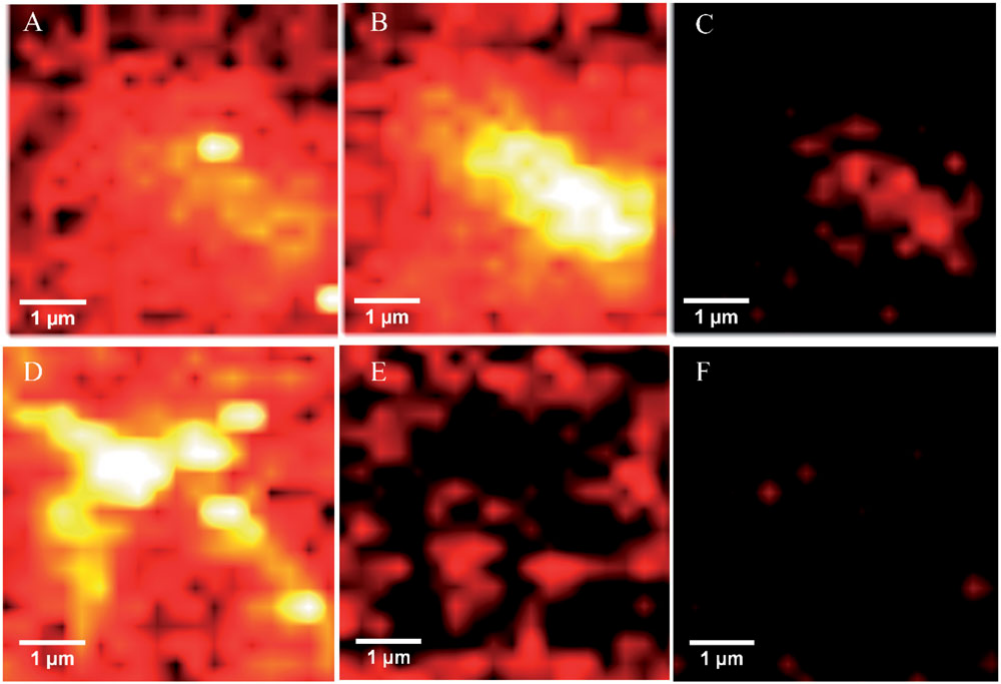
Upper panels: Raman maps of tau aggregation in the absence of **5** showing intensities of distinct Raman’s peaks. Images were generated from the same sample region by using the sum filter (integral) over a small window of normal modes: (A) C–H deformation 1451 cm^−1^ (B) Amide I-1670 cm^−1^) (C) Amide III-1240 cm^−1^. Lower panels: Raman maps taken in the same sample region) of tau aggregation in the presence of **5**. (D) C–H deformation 1451 cm^−1^. (E) Amide I-1670 cm^−1^. (F) Amide III-1240 cm^−1^.

Raman maps based on the integral of different Raman bands give the relative intensities of defined peaks in the spectral region. Thus, we analyzed peaks associated to β sheets (Amide I and III), and C–H deformation. As we compared aggregation control, with samples treated with **5**, there are differences between peak intensities, including Amide I (1670 cm^−1^) (Figure 6(B,D)), and Amide III (1240 cm^−1^) (Figure 6(C,E), respectively), indicating that relative intensities associated to β sheets are also diminished. However, once we compare C–H deformation (1451 cm^−1^) in both controls and treated sample, significant differences were not found (Figure 6(A,D)), indicating that sample amounts remain similar in both (control and treated) once we scan over the glass surface to determinate Raman spectra.

### Molecular modeling of 5 binding to tau’s ^306^VQIVYK^311^ hexapeptide

Tau is considered a natural unstructured protein^16^, although there is limited information on its exact structure. However, the structure of the fibril-forming hexapeptide motif of tau, ^306^VQIVYK^311^, has been resolved by X-ray crystallography in complex with orange-G (Protein Data Bank code 3OVL)^9^.

Orange-G is bound between steric zippers of VQIVYK with its long axis parallel to the fiber axis and forms salt links between its sulfonic acid groups and lysine ammonium ions from repeating strands in VQIVYK fibers. In addition, the aromatic rings of orange-G are packed against apolar side chains of Val309, and establish polar interactions with glutamine and lysine side-chains at the edges of the steric zipper. Recent works have used the information of the VQIVYK-orange-G crystals to propose interactions between ligands that prevent tau aggregation and the hexapeptide VQIVYK using docking method^55^^,^^61^.

Compound **5** has an elongated shape, aromatic and polar groups, and a negatively charged group, which match with the chemical features orange-G. With this in mind, we propose that rosmarinic acid inhibits tau aggregation by establishing chemical interactions with the fibril-forming hexapeptide VQIVYK.

**Figure 7.**
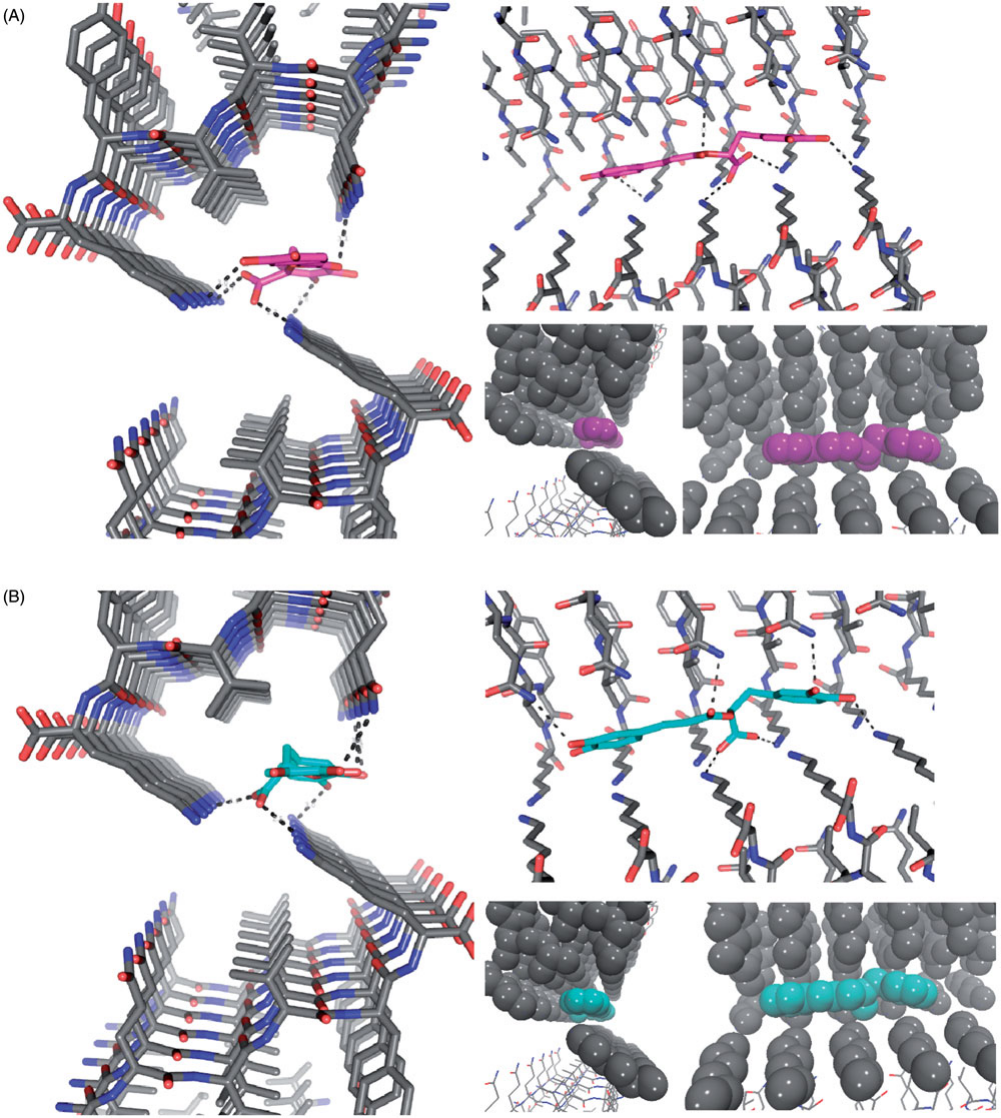
The structure of the ^306^VQIVYK^311^ segment from tau protein complexed with rosmarinic acid obtained by docking in the interface of two zippers. The VQIVYK β-sheets segments are packed in parallel forming two steric zippers. The Figure shows **5** mediates contacts between two pairs of steric zippers. (A) R enantiomer, (B) S enantiomer. In both A and B: *left*: view looks down the fiber axis; *right-top*: the view is perpendicular to the fiber axis. HBs and ionic interactions between ligand carboxylate and lysine amines are represented with dashed lines; *right-bottom*: ligand and VQIVYK carbon atoms are represented as balls to observe apolar packing.

To obtain a better understanding of a possible interaction of **5** with the ^306^VQIVYK^311^ motif in 4R oligomers, we model **5** binding (R and S enantiomers were considered) to β-sheets of poly-^306^VQIVYK^311^ hexamers. X-ray crystallography revealed that fiber-like complexes of ^306^VQIVYK^311^ consist of pairs of β-sheets, allowing small molecules to bind between sheets^9^. In particular, negatively charged molecules bound specifically to ^306^VQIVYK^311^ lysine side chains of adjacent sheets. Rosmarinic acid docking was done inside the cylindrical cavity formed by paired ^306^VQIVYK^311^ β-sheets (Figures 7(A,B)).

Our docking models depict that both **5** enantiomers are placed inside the cavity without any steric clashes (Figure 7(A,B)). According to our results, **5** and ^306^VQIVYK^311^, chemical interactions are comparable to those observed for orange G^9^. Both rosmarinic acid enantiomers have the aromatic rings packed against apolar side chains of Val309 and form a hydrogen bond (HB) network with glutamine and lysine side chain groups at both sides of the steric zipper. The most important interaction is the salt link between the carboxylate group of rosmarinic acid and two Lys311 ammonium ions from ^306^VQIVYK^311^ fibers.

Taken together, our docking models predict a possible interaction of **5** with ^306^VQIVYK^311^ β-sheets with chemical interactions comparable to the ones reported for orange G. Therefore, we suggest that anti-aggregation effects of **5** are due to interactions with ^306^VQIVYK^311^, in concordance with other reports that suggested the same mechanism for other known drugs.

## Conclusions

In the present study, we isolated five compounds **1**–**5** from *R. officinalis* L. All of them demonstrated to inhibit tau aggregation over 50% at 50 μM. Moreover, we found that **5** was the most active compound. In addition, we found that both morphology and content of β sheet is affected once we challenged tau with **5**. Docking experiment revealed that **5** interact with ^306^VQIVYK^311^ mainly through salt bridges. Since there is only palliative treatment against Alzheimer’s disease and tauopathies, we consider important to search for promising naturally compounds as potential drug pharmacophore with catechol moities.
